# Minimally Invasive Pectus Excavatum Correction and Endoscopic Port Access Mitral Valve Surgery

**DOI:** 10.5041/RMMJ.10517

**Published:** 2024-01-19

**Authors:** Johan van der Merwe, Filip Casselman, Ivan Degrieck, Frank Van Praet

**Affiliations:** 1 The Keyhole Heart Centre, Netcare Blaauwberg Hospital, Cape Town, South Africa; 2 Cardiovascular Surgery, Cardiovascular Centre, OLV Clinic, Aalst, Belgium

**Keywords:** Minimally invasive cardiac surgery, mitral and tricuspid valve disease, pectus excavatum

## Abstract

This case study describes the successful short-term outcome of staged minimally invasive pectus excavatum correction and endoscopic mitral valve repair in a patient with severe mitral valve regurgitation and pectus excavatum.

## INTRODUCTION

Contemporary minimally invasive atrioventricular valve surgery (MI-AVVS) that utilizes direct or endoscopic vision, special single-shaft instruments, or innovative robotic technology is well established.^1^ Adult patients with indications for MI-AVVS and uncorrected congenital chest wall deformities (CCWD), which include the spectrum of isolated and mixed pectus deformities, present unique intraopera-tive challenges in obtaining adequate valve exposure and unobstructed working angles.^2^ Previous reports on how to address these challenges during MI-AVVS have described the use of preoperative computerized tomography to plan minimally invasive atrial retrac-tor and working port positioning^3^ and single-stage correction of the CCWD by a modified conventional Ravitch procedure preceding MI-AVVS.^4^ We per-formed 90 Nuss procedures^5^ since the initiation of our program in 2008, which is well described and regarded as an excellent less invasive alternative for pectus excavatum (PE) correction.^6^ Our endoscopic MI-AVVS program was established in 1997^7^ and includes 3,451 procedures up to May 31, 2023. We describe the successful short-term outcome of staged minimally invasive PE correction and endo-scopic mitral valve repair in a patient with severe mitral valve regurgitation and PE.

## CASE REPORT

**Figure 1:**
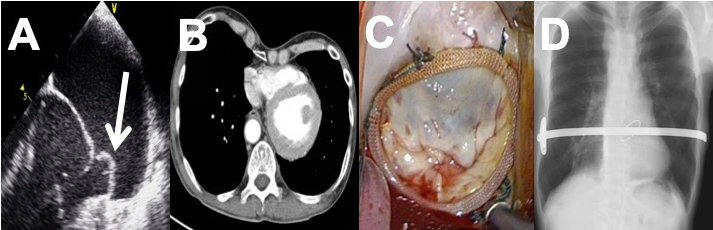
Perioperative and Procedural Images of Staged Endoscopic Mitral Valve Repair and Pectus Excavatum Correction Surgery **A:** Trans-thoracic echocardiography indicating a prolapsing P2 mitral valve segment (white arrow). **B:** Computerized tomography indicating cardiac displacement to the left hemithorax. The Haller Index* and the Correction Index^†^ were calculated to be 3.1 and 33.9%, respectively. **C:** Endoscopic mitral valve repair. **D:** Post-operative chest X-ray indicating satisfactory Nuss bar positioning. *Haller Index: the ratio of the transverse thoracic (25.4 cm) and minimum sternovertebral diameter (7.6 cm). ^†^Correction Index: the indentation depth (3.9 cm) as a percentage of the maximum sternovertebral diameter (11.5 cm).

The echocardiogram of a 43-year-old male with progressive New York Heart Association (NYHA) class III symptoms confirmed severe degenerative mitral valve regurgitation due to a posterior leaflet chordal rupture (Figure 1A). He underwent an aortic vascular ring correction at the age of 1 year through a left thoracotomy and sustained a subsequent per-manent left recurrent laryngeal nerve paralysis. Our institutional multidisciplinary team favored surgical mitral valve intervention and additional investiga-tions, which included coronary and aorta-iliac axis angiography, thoracic computerized tomography, electrocardiography, and lung function tests; these were all uneventful. The Haller Index (defined as the transverse thoracic and minimum sternovertebral diameter ratio^8^) and the Correction Index (described as the indentation depth as a percentage of the maximum sternovertebral diameter^9^) were 3.1 and 33.9%, respectively, as determined by the preopera-tive chest computerized tomography (Figure 1B).

The patient elected to have a staged endoscopic assisted Nuss and mitral valve procedure with a cal-culated EuroSCORE II of 3.46. The minimally inva-sive PE correction was performed first. Intraopera-tive sternocostal defect measurements and Nuss bar preparation (Biomet, 36 cm, Wilrijk, Belgium) fol-lowed routine double lumen endotracheal intuba-tion and endoscopic camera port placement (5.5 mm, Olympus, Hamburg, Germany) in the fourth intercostal space, mid-axillary line. The pre-shaped Nuss bar was positioned through a right 2.5 cm anterior-axillary line incision after an introducer de-vice and tape created an endoscopically guided retro-sternal tract to the left hemithorax. A stabilizing metal plate anchored the bar on the right, and resorbable sutures secured the bar through a 2 cm incision on the left. The camera port facilitated the insertion of an intrathoracic drain (Redon CH 8, PMF Medical, Köln, Germany), and the patient was transferred to intensive care for routine analgesia and monitoring after uneventful intraoperative extubation.

Our MI-AVVS technique is well described^10^ and was performed after an interprocedural time inter-val of 3 days. Routine cardiac anesthesia, which in-cluded single-lung ventilation, was followed by trans-esophageal echocardiographic-guided cannulation of the right internal jugular vein (18Fr, Optisite™, Edwards Lifesciences, Irvine, CA, USA), femoral vein (25Fr, Quickdraw™, Edwards Lifesciences), and right femoral artery cannula (23Fr, Endoreturn™, Edwards Lifesciences). An endo-aortic balloon (IntraClude™, Edwards Lifesciences) was used for aortic occlusion and cold antegrade crystalloid car-dioplegia delivery. The camera port used in the PE correction was re-utilized as a 4 cm working port in the fourth anterior-axillary intercostal space, with easy visualization of the Nuss bar and cardiac structures. The left atrial retractor was positioned in the right parasternal fourth intercostal space. The initiation of cardiopulmonary bypass and subsequent endo-aortic balloon occlusion were uneventful, and access to the mitral valve was unrestricted and easily established. Systematic valve analyses concurred with preoperative imaging findings, and a successful endoscopic mitral valve repair was performed (Fig-ure 1C) using long-shafted instruments that consist-ed of annular ring implantation (CE Physio II™, size 40, Edwards Lifesciences), quadrangular resection of the prolapsing P2 segment, and neochordal attachment of the posterior leaflet to the posterior-medial papillary muscle (Gore-Tex™, Gore & Asso-ciates Inc., Phoenix, AZ, USA). Deairing was en-sured by a venting catheter in the left atrium, ante-grade balloon catheter venting, and transesophageal echocardiogram surveillance for residual air in the left ventricle. Cardiopulmonary bypass and ischemic times were 164 min and 97 min, respectively, with extubation achieved 6 hours postoperatively.

Rapid patient recovery resulted in home dis-charge after 14 days despite urgent re-intubation for acute airway obstruction related to his premorbid laryngeal nerve paralysis on the second postopera-tive day following cardiac surgery. Predischarge echocardiography confirmed a satisfactory mitral valve repair and the absence of any residual mitral valve regurgitation. Follow-up at 6 weeks revealed excellent clinical, cosmetic, radiological, and echo-cardiographic recovery (Figure 1D).

## DISCUSSION

Pectus excavatum is reported to develop in 0.3% of the general population and is characterized by the posterior depression of the lower costal cartilages and sternum.^5^ Patients with uncorrected CCWD that include the spectrum PE may require atrioven-tricular valve surgery.^3^ The decreased anterior-posterior thoracic diameter, cardiac displacement to the left hemi-thorax, and compression of the right ventricle are amongst the anatomical features that present difficult access and potentially restricted working angles in MI-AVVS.

We previously reported our MI-AVVS experience in seven consecutive patients with decreased antero-posterior and sternovertebral diameters, which in-cluded Haller and Correction indices of up to 3.3 and 38.3%.^3^ No sternotomy conversions, revisions, stroke, renal dysfunction, wound complications, 30-day or long-term mortalities, or reinterventions were observed over a mean follow-up of 29.7±26.5 months (range 0.2–72.2). We concluded that MI-AVVS in PE was safe, feasible, and durable, with favorable long-term clinical, radiological, and echo-cardiographic outcomes when performed in experi-enced centers. However, even though adequate atrioventricular valve exposure was obtained in all patients, we acknowledged that extreme PE may prohibit MI-AVVS.

The combination of staged endoscopically en-hanced Nuss and MI-AVVS corrects the external chest wall morphology, facilitates unrestricted intra-cardiac access for standard valve repair and replace-ment, and offers the full range of benefits associated with both minimally invasive procedures. We rou-tinely utilize unilateral 23F or 21F femoral artery Y-arm cannulation that facilitates endo-aortic balloon access, with a low threshold to cannulate the contra-lateral femoral artery (17F or 19F) to ensure optimal flow and perfusion.^10^ The aorta-iliac axis is evalu-ated preoperatively by contrasted CT or additional contrast injection during coronary angiography.

The principles of mitral valve repair are well established,^11^ and we elected to perform a conserva-tive resection of excessive posterior leaflet tissue and to ensure perfect coaptation with the addition of neochords. Even though PE correction and MI-AVVS in a single operative setting may be attractive, we elected to stage the procedures to minimize the potential risks of Nuss bar insertion tract bleeding secondary to systemic heparinization required for cardiopulmonary bypass. No reports currently describe the intraoperative conversion strategies for MI-AVVS in patients with Nuss bars *in situ*.

We previously described our reasons for sternot-omy conversion and adverse intraoperative events in 2,872 MI-AVVS procedures,^12^ and the presence of a Nuss bar presents unique conversion challenges. Extensive experience in both MI-AVVS and Nuss procedures are regarded as prerequisites to our described staged procedures and should not be part of the initial learning experiences. Possible options include the extension of the working port to a larger thoracotomy, the rapid removal of the bar and sub-sequent midline sternotomy, and conversion to hemi-clamshell/clamshell access with its well de-scribed complications and morbidities.^13^ The pro-longed hospitalization of 14 days was not procedure-related.

This report demonstrates the feasibility of com-bining minimally invasive Nuss and MI-AVVS pro-cedures as a high-quality alternative to conventional Ravitch and sternotomy approaches for atrioven-tricular valve procedures in CCWD patients.

## Abbreviations

CCWD: congenital chest wall deformities
MI-AVVS: minimally invasive atrioventricular valve surgery
NYHA: New York Heart Association
PE: pectus excavatum
